# The State of the World’s Midwifery 2021 report: findings to drive global policy and practice

**DOI:** 10.1186/s12960-021-00694-w

**Published:** 2021-11-27

**Authors:** Andrea Nove, Petra ten Hoope-Bender, Martin Boyce, Sarah Bar-Zeev, Luc de Bernis, Geeta Lal, Zoë Matthews, Million Mekuria, Caroline S. E. Homer

**Affiliations:** 1 Novametrics Ltd, Duffield, DE56 4HQ UK; 2 UNFPA, 7 rue de Varembé, 1202 Geneva, Switzerland; 3 UNFPA, 605 Third Avenue, New York, NY 10158 USA; 4 University of Southampton, Southampton, SO17 1BJ UK; 5 Burnet Institute, 85 Commercial Road, Melbourne, VIC 3004 Australia

**Keywords:** Midwives, Midwifery, Human resources for health, Health workforce, Sexual, reproductive, maternal, newborn and adolescent health

## Abstract

The third global State of the World’s Midwifery report (SoWMy 2021) provides an updated evidence base on the sexual, reproductive, maternal, newborn and adolescent health (SRMNAH) workforce. For the first time, SoWMy includes high-income countries (HICs) as well as low- and middle-income countries. This paper describes the similarities and differences between regions and income groups, and discusses the policy implications of these variations. SoWMy 2021 estimates a global shortage of 900,000 midwives, which is particularly acute in low-income countries (LICs) and in Africa. The shortage is projected to improve only slightly by 2030 unless additional investments are made. The evidence suggests that these investments would yield important returns, including: more positive birth experiences, improved health outcomes, and inclusive and equitable economic growth. Most HICs have sufficient SRMNAH workers to meet the need for essential interventions, and their education and regulatory environments tend to be strong. Upper-middle-income countries also tend to have strong policy environments. LICs and lower-middle-income countries tend to have a broader scope of practice for midwives, and many also have midwives in leadership positions within national government. Key regional variations include: major midwife shortages in Africa and South-East Asia but more promising signs of growth in South-East Asia than in Africa; a strong focus in Africa on professional midwives (rather than associate professionals: the norm in many South-East Asian countries); heavy reliance on medical doctors rather than midwives in the Americas and Eastern Mediterranean regions and parts of the Western Pacific; and a strong educational and regulatory environment in Europe but a lack of midwife leaders at national level. SoWMy 2021 provides stakeholders with the latest data and information to inform their efforts to build back better and fairer after COVID-19. This paper provides a number of policy responses to SoWMy 2021 that are tailored to different contexts, and suggests a variety of issues to consider in these contexts. These suggestions are supported by the inclusion of all countries in the report, because it is clear which countries have strong SRMNAH workforces and enabling environments and can be viewed as exemplars within regions and income groups.

**Supplementary Information:**

The online version contains supplementary material available at 10.1186/s12960-021-00694-w.

## Introduction

Despite the significant progress over the past two decades in improving outcomes for sexual, reproductive, maternal, newborn and adolescent health (SRMNAH), progress has been uneven. Maternal mortality, neonatal mortality and stillbirth rates remain high in many countries, a large number of women give birth without assistance from a skilled health provider, there is a considerable amount of unmet need for modern contraception, and quality of care is often suboptimal [1].

Resilient health systems grounded in primary health care are vital to the health and well-being of every woman, newborn and adolescent. The COVID-19 pandemic has highlighted the importance of resilient health systems, especially health workforces. The Global Strategy on Human Resources for Health stresses that without an effective health workforce no health system is viable and universal health coverage cannot be achieved [2]. High-quality SRMNAH care requires a competent, educated, motivated and well supported workforce. Improving SRMNAH requires increased commitment to, and investment in, the health workforce.

The third global *State of the World’s Midwifery* (SoWMy) report was published in May 2021 by the United Nations Population Fund (UNFPA), the International Confederation of Midwives (ICM) and the World Health Organization (WHO), to provide an updated evidence base and detailed analysis of the progress and challenges to delivering effective coverage of high-quality midwifery services [1]. The first two SoWMy reports in 2011 and 2014 [3, 4] led to some substantial advances, political commitments and achievements in a number of countries [5]. However, more needs to be done as a matter of urgency: Sustainable Development Goals (SDGs) 3 and 5 will not be met by 2030 without increased commitment to and investment in the education, recruitment, deployment, retention and management of midwives and other SRMNAH workers.

Just prior to the publication of the main SoWMy report, the SoWMy team published a study which concluded that universal coverage of a set of essential “midwife-delivered interventions” (i.e., which are known to be linked to lower mortality rates and which can be delivered in their entirety by a midwife educated to global standards who is working within an enabling environment) could avert approximately two-thirds of the world’s maternal and neonatal deaths and stillbirths, saving over four million lives per year by 2035 [6].

SoWMy 2011 and SoWMy 2014 focused exclusively on the low- and middle-income countries with the highest rates of maternal and neonatal mortality, whereas all 194 WHO Member States were eligible for inclusion in the 2021 report. The objective of this paper is to describe the observed similarities and differences between different regions and income groups, and to discuss the policy and strategy implications of these variations.

## SoWMy 2021 approach and key findings

The two main data reporting mechanisms for SoWMy 2021 were: the WHO National Health Workforce Accounts (NHWA) platform [7] and the ICM Global Midwives Associations Map Survey [8]. The NHWA platform, established in October 2017 as the WHO official reporting system for health workforce statistics, is updated on an ongoing basis with government-validated data that have been checked for consistency. The ICM survey was completed in 2019–2020 by professional midwife associations or UNFPA country offices, and validated by the competent national authorities. Full details of the methods used have been published elsewhere [9].

The analysis uses three key concepts to measure workforce availability and accessibility: (i) “need”, defined as the amount of health worker time that would be required to achieve universal coverage of a set of essential SRMNAH interventions, (ii) “supply”, defined as the amount of SRMNAH worker^1^ time available to spend on SRMNAH interventions, and (iii) “demand”, defined as the economic capacity of a country to employ health workers.

In relation to “need”, SoWMy 2021 estimates that in 2019, approximately 6.5 billion health worker hours were required to meet all of the world's need for essential SRMNAH interventions. Just over half of these hours (55%) are for maternal and newborn interventions, 8% for adolescent sexual and reproductive health (SRH) interventions, and the remaining 37% for other SRH interventions such as contraception and sexually transmitted infections. The workforce must, therefore, have the competencies to meet a wide variety of SRMNAH needs across the life course in addition to pregnancy and childbirth interventions.

SoWMy 2021 estimates a global shortage of 900,000 midwives. If current trends of increased supply continue, this is projected to decrease only slightly (to 750,000) by 2030. It also estimates that the current SRMNAH workforce cannot meet more than 75% of the world’s need for essential SRMNAH interventions. In reality, it is likely that the workforce meets far less than 75% of the need, due to issues, such as: geographical maldistribution, poor working environments, the costs of accessing care, weak commodity supply chains, and gaps in quality of care due to, *inter alia*, poor quality education and regulation, and gender discrimination in the workforce.

SoWMy 2021 projects estimates of “demand” forward to 2030 and predicts that most countries will have a mismatch between the supply of SRMNAH workers and the number the country can afford to employ. About half of countries will have a demand-based shortage, i.e., they will produce fewer SRMNAH workers than their economy can support, and about half will have a demand-based oversupply, i.e., they will produce more than their economy can support (assuming spending priorities remain unchanged).

Investing in midwives can clearly yield important returns, including: more positive birth experiences, improved health outcomes, inclusive and equitable economic growth. Although the causal mechanism for these improved outcomes is not clear, SoWMy 2021 suggests that it is related to the unique philosophy of midwifery which takes a life-course approach and focuses on woman-centred, preventive, supportive care within a functioning referral system should medical intervention be required.

The impact of COVID-19 on the midwifery workforce is still being evaluated, but it is clear that many midwives and other SRMNAH workers were not sufficiently protected from infection and lost their lives due to the virus (SoWMy 2021 is dedicated to them), and many more are suffering from burnout, exhaustion and trauma. Health systems worldwide need to plan for replacing the losses and supporting the remaining health workforce to stay in post and provide high-quality care. This provides an opportunity to make improvements to SRMNAH care via strategic investments in the workforce. The dependence of the SRMNAH workforce on women (SoWMy 2021 reported that 93% of midwives, 89% of nurses and 50% of SRMNAH doctors^2^ are women) means that a gender-transformative approach is needed, to address the gender-related challenges encountered by women in the health workforce.

**Fig. 1:**
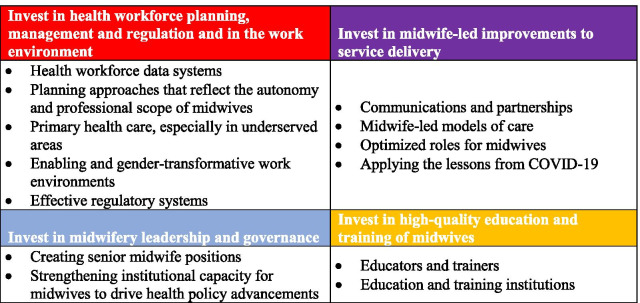
Summary of investments needed for midwifery

To help bring about this transformation and develop an SRMNAH workforce that is sufficiently large, qualified and supported to meet all of the need for high-quality care, SoWMy 2021 calls for investment in four areas (Fig. 1).

## Income group patterns

The SoWMy 2021 analysis is based on country income group classifications as they were in November 2020 (Additional file 1: Table S2 provides a list of countries in each income group). It provides clear evidence of a major mismatch between the need for midwives, nurses and doctors and the overall supply. High-income countries (HICs) account for 11% of the need and 41% of the supply, whereas low-income countries (LICs) account for 14% of the need and 2% of the supply.

For nurses and doctors, the pattern is the same: on average, HICs have the highest density,^3^ followed by upper-middle-income countries (UMICs), then lower-middle-income countries (LMICs), then LICs. For midwives, however, the density is higher in middle-income countries than in HICs. The figures are skewed by Indonesia: a large middle-income country with many midwives, but even if Indonesia is excluded, midwife density is similar in HICs and middle-income countries. In other words, the greater supply of SRMNAH workers in HICs is largely due to their having more doctors and nurses.

LICs tend to have the lowest density of all three types of SRMNAH worker, but on average, a quarter of the available SRMNAH worker time is from midwives, compared with less than 10% in HICs. In LMICs, the percentage is even higher: about a third of the available SRMNAH worker time is from midwives. However, a large majority of the midwives in LMICs are classed as associate professionals rather than professionals (see notes under Additional file 1: Table S1 for details), implying that their range of skills and competencies is relatively limited.

Mapping of supply against need in the SoWMy 2021 report shows that HICs have sufficient SRMNAH workers to meet all of the need, and that UMICs have enough to meet most of the need. Needs-based shortages are most severe in LICs but also evident in LMICs: three-quarters of the global shortage of 900,000 midwives comes from LICs and LMICs.

Based on current trends, most of the projected growth in supply to 2030 is expected to occur in LMICs, rather than in LICs, where the shortage is most profound. This pattern is emphasized by the SoWMy 2021 analysis of the extent to which supply in 2030 will match economic demand. About half of all countries are projected to produce fewer SRMNAH workers than they can afford to employ (i.e., they will have a demand-based shortage), but nearly all of the countries projected to have a *severe* demand-based shortage are LICs and LMICs.

The education and regulatory environment for midwives tends to be stronger in HICs and UMICs than in LMICs and LICs. For example, HICs and UMICs are more likely to: offer midwife education programmes which meet ICM recommendations for duration of course, have midwives educating midwives, offer postgraduate study in midwifery, and have legislation and regulatory systems which recognise midwifery and nursing as distinct professions. Most HICs have laws/policies for the prevention of physical or verbal attacks on health workers, compared to only about half of UMICs, LMICs and LICs.

However, other indicators of the strength of the midwifery profession reveal relative strengths in LICs and LMICs. For example, the percentage of LICs with a professional association specifically for midwives is similar to the percentage in HICs and UMICs. Most UMICs and LICs reported at least one midwife in a leadership position within the national ministry of health (MoH),^4^ compared with just one in five HICs. Midwives in LICs and LMICs tend to have a broader scope of practice, with far fewer restrictions to the number of basic emergency obstetric and newborn care (BEmONC) signal functions and contraceptive methods which they can provide.

## Regional patterns

Inequity between the need for and availability of SRMNAH workers is also evident between WHO regions (see Additional file 1: Table S3 for a list of countries in each region). SoWMy 2021 shows that Africa and South-East Asia account for half of the world’s need for SRMNAH worker time, but just 20% of the world’s midwives, nurses and doctors. By contrast, Europe and the Americas account for 20% of the need but 50% of the supply.

Although *Africa* has the lowest density of SRMNAH workers overall, relative to the overall size of the workforce this region has the highest proportion of professional midwives, and nearly 40% of the available SRMNAH worker time is from midwives. Africa stands out as having the most severe SRMNAH worker shortage in the world: it accounts for over half of the global shortage, the workforce can meet no more than half of the need, and in reality it almost certainly meets much less than half. These challenges, coupled with rapid population growth in many African countries, mean that the situation is predicted to improve only slightly by 2030 unless there is significant additional investment.

Midwives in Africa tend to have a broader scope of practice than those in other regions: they are generally authorized to perform all seven BEmONC signal functions and provide all modern methods of contraception. Africa also has a high proportion of countries with midwife leaders in the national MoH: it is second only to the Americas on this indicator. About half of responding countries in this region offer postgraduate study in midwifery. On the other hand, many African countries rely on midwife educators who are not themselves midwives to teach pre-service education programmes, indicating a shortage of suitably qualified midwives to teach the next generation. Fewer than half of African countries report that their midwives must provide evidence of continuing professional development (CPD), which calls into question whether the skills of the midwifery workforce are routinely kept up-to-date.

The *Americas* is the region with the lowest midwife density and the highest nurse density: indicating that this region relies very heavily on nurses as providers of SRMNAH interventions. Fewer than half of responding countries in this region offer a postgraduate qualification in midwifery. Despite this, most responding countries report that midwifery is recognised as a separate profession, that it has a separately regulatory system, that there is a professional association specifically for midwives and that there are midwife leaders in the national MoH.

The *Eastern Mediterranean* region has the second lowest SRMNAH worker density after Africa and accounts for almost 20% of the global midwife shortage. Its SRMNAH workforce can meet no more than 70% of the need (and like Africa, probably meets much less than this). In contrast to Africa, however, current trends suggest that the situation will be much improved by 2030. Most of the midwives in the Eastern Mediterranean region are professionals, but most of its nurses are associate professionals. Relative to the number of midwives and nurses, the region has a lot of doctors in its SRMNAH workforce, indicating a medicalized SRMNAH care system in many countries in the region.

The Eastern Mediterranean region is one of only two regions, where the vast majority of midwife educators are themselves midwives (the other being Europe). However, fewer than half of countries in this region offer a postgraduate qualification in midwifery, fewer than half have a separate regulation system for midwives and only half require midwives to provide evidence of CPD to continue practising. Some countries in the region restrict the midwife’s scope of practice, e.g., fewer than half of countries authorize midwives to conduct manual placenta removal and manual vacuum aspiration.

The overall density of SRMNAH workers in *Europe* is similar to the Americas, but midwife density is 2.5 times higher in Europe than in the Americas, and nearly all of Europe’s midwives are professionals rather than associate professionals. As noted above, most European countries use midwives to educate midwives, and it is the only region in which the majority of countries offer a postgraduate qualification in midwifery. Similarly, nearly all countries have a separate regulatory system for midwives and an association specifically for midwives. On the other hand, very few countries in this region have a midwife leader in the national MoH and the scope of practise of midwives is often restricted, e.g., very few countries permit midwives to perform vacuum extraction and manual vacuum aspiration, and midwives do not tend to be authorized to provide modern contraceptives.

A large proportion of the SRMNAH workers in *South-East Asia* are midwives, but nearly all of this region’s midwives are associate professionals rather than professionals and, therefore, can provide a smaller number of essential SRMNAH interventions. About half of responding countries in this region offer a postgraduate qualification in midwifery. The scope of practise of midwives is broader than in all other regions except Africa. However, only about half of the reporting countries in this region have legislation recognizing midwifery as distinct from nursing, only about two-thirds have a separate regulatory system for midwives, and fewer than one in three have a midwife leader in the national MoH.

The *Western Pacific* region has a relatively high midwife density, second only to South-East Asia, and nearly all of its midwives are professionals. However, in this region, less than 10% of the available SRMNAH worker time comes from midwives, because there is an even higher density of nurses and doctors. Nearly all responding countries in this region have a separate regulatory system for midwives and a professional association specifically for midwives, and the scope of practice of a midwife tends to be broad (the main exception being that fewer than half of countries in this region authorize midwives to perform manual vacuum aspiration). However, fewer than half of countries offer a postgraduate qualification in midwifery, fewer than half have midwife leaders in the national MoH, and fewer than half require periodic evidence of CPD.

## Limitations

Although all 194 WHO Member States submitted at least one data item to the SoWMy 2021 report, no country provided validated data for all of the SoWMy 2021 indicators. For several indicators the number of responding countries was well below 100. Therefore, it is possible that some of the regional and income group results presented in SoWMy 2021 and described in this paper do not accurately represent the situation in that region or income group. This particularly affects the indicators relating to midwife education, regulation and scope of practice, three critical issues for midwifery development and maternal and perinatal health. There is an urgent need for better data availability and quality in many countries. The SoWMy 2021 webappendices [9] provide details of which countries provided data for each indicator.

The regional and income group estimates for health worker numbers and density are weighted by population size, so larger countries have a stronger impact on the average than smaller countries. While not a limitation as such, it is important to bear this in mind when interpreting the figures. Furthermore, regional and income group averages can mask important variations between and within countries. Within each region and income group, there will be countries which are typical of the group of countries within it, and others which are not.

## The policy implications of SoWMy 2021

For the first time in the history of SoWMy, the 2021 report is truly global and, therefore, includes a wider range of analyses, including a new method of estimating demand-based workforce shortages (or oversupplies) as well as estimating needs-based shortages. This makes the report relevant to all countries, and in this paper we have attempted to highlight this broad applicability. It also allows us to draw conclusions about how the policy implications of the data in the report may be different in different contexts.

The main stories from the data on SRMNAH workforce supply are the major global shortage of SRMNAH workers and the inequity between higher and lower income countries—in particular the urgent need to address the severe shortages in LICs and in the African region to achieve the health-related SDGs. Many LICs are projected still to have severe needs-based SRMNAH worker shortages in 2030. It should be noted that the needs-based shortage estimates in SoWMy 2021 are separate from the demand-based shortage estimates: it is possible for a country to have neither type of shortage, just one type, or both types. A needs-based shortage brings with it an imperative to increase production of SRMNAH workers. However, if this is coupled with a demand-based oversupply, then increased production is likely to lead to ‘brain drain’ if the country does not have the fiscal space to employ all of the SRMNAH workers it produces. Many LICs are in this situation, which means efforts are required both to increase the supply of SRMNAH workers and to boost market-based demand to ensure that the increased supply of qualified individuals can get jobs in the health sector.

Even among HICs and UMICs, very few countries are predicted to achieve a good match between supply and economic demand, which implies the need for better workforce planning systems across the board.

Some of the SoWMy 2021 indicators relating to quality of care indicate widespread systemic issues. For example, the only region in which most countries offer a postgraduate qualification in midwifery is Europe, which perhaps partly explains why so many LMICs and LICs in other regions rely on other types of health professional to teach midwives. Stronger midwifery departments in universities would encourage further study and research on midwifery, and encourage midwives to take the lead in the education and research which is greatly needed. Similarly, in most regions fewer than half of countries have a requirement for periodic proof of CPD as part of midwifery regulation. Coupled with the well-documented weaknesses in midwife pre-service education in many countries [10], this means that many of the world’s midwives may lack the competencies to provide high-quality care.

The dependence in HICs and UMICs—especially in the Americas region—on doctors and nurses to provide SRMNAH interventions raises questions about whether the lower mortality rates for women and newborns observed (on average) in these settings come at the expense of access to the unique philosophy and model of care provided by midwives, which has been shown to facilitate positive birth experiences and improve other types of health outcomes. This concern is underlined by the finding that the scope of practice of midwives tends to be more restricted in HICs and UMICs (especially in Europe and the Eastern Mediterranean) than in LMICs and LICs. SoWMy 2021 points out that midwives who are educated and regulated according to global standards can meet around 90% of the need for essential SRMNAH interventions, but this is achievable only if they are authorized to operate to their full scope of practice within an enabling work environment [11].

Similarly, the heavy reliance in some LMICs on associate professional midwives calls into question their ability to provide midwifery services across the full continuum of care, so the high density of midwives in LMICs—especially those in South-East Asia—may not translate to widespread access to high quality midwifery care. By contrast, nearly all of Africa’s midwives are classed as professionals, perhaps indicating a greater appreciation of the potential of midwives to meet most of the need for essential SRMNAH interventions, even if currently this region does not have anywhere near enough of them and quality of care can still be poor.

Addressing the issues highlighted in SoWMy 2021 requires strong leadership for the midwifery profession, focusing on the four areas of investment shown in Fig. 1. Yet only half of countries have midwife leaders in the national MoH, and the proportion is much lower than this in HICs, especially in Europe and South-East Asia. The creation of leadership roles for midwives, and the inclusion of midwives in candidate lists for existing leadership roles, will be important to ensure that appropriate investments in midwifery are made.

## Conclusion

Since the first SoWMy report in 2011, there has been much progress in midwifery, including greater recognition of the importance of quality of care, widespread accreditation systems for health worker education institutions, and greater recognition of midwifery as a distinct profession. On the other hand, many of the issues highlighted in the two previous SoWMy reports remain of concern, such as workforce shortages, an inadequate working environment, low-quality education and training, and limitations in health workforce data.

Governments and relevant stakeholders are urged to use SoWMy 2021 to inform their efforts to build back better and fairer from the COVID-19 pandemic, forging stronger primary health-care systems as a pathway to UHC and fostering a more equitable world. It is hoped that the pandemic will be a catalyst for change given the heightened profile of health workers. SoWMy 2021 can help make this happen.

## Supplementary Information

**Additional file 1: Table S1.** Health occupations classed as part of the SRMNAH workforce. **Table S2.** List of WHO Member States by World Bank income group, 2020. **Table S3.** List of WHO Member States by WHO region, 2020.

## Data Availability

The data sets analysed for the SoWMy 2021 report are available from the National Health Workforce Accounts platform (https://apps.who.int/nhwaportal/) and the ICM Midwives Hub platform (https://www.globalmidwiveshub.org/).

## Abbreviations

BEmONC: Basic emergency obstetric and newborn care
CPD: Continuing professional development
HIC: High-income country
ICM: International Confederation of Midwives
LIC: Low-income country
LMIC: Lower-middle-income country
MoH: Ministry of Health
NHWA: National Health Workforce Accounts
SDGs: Sustainable development goals
SoWMy: State of the World’s Midwifery
SRH: Sexual and reproductive health
SRMNAH: Sexual, reproductive, maternal, newborn and adolescent health
UMIC: Upper-middle-income country
UNFPA: United Nations Population Fund
WHO: World Health Organization

## Footnotes

1 SoWMy 2021 used International Standard Classification of Occupations codes to define the SRMNAH workforce—see Additional file 1: Table S1 for details.

2 SoWMy 2021 uses the term “SRMNAH doctors” to refer to general practitioners, obstetricians/gynaecologists and paediatricians: the three types of doctor most likely to provide SRMNAH interventions.

3 Health worker density is the number of health workers per head of population.

4 The phrase “leadership position” was defined as referring to a number of management, supervisory and executive titles, including: Chief Midwife, Midwife Advisor, national Midwife Director, maternal advisory position.
