# Supplementary material for: The State of the World’s Midwifery 2021 report: findings to drive global policy and practice
Source: Hum Resour Health. 2021 Nov 27;19:146. doi: 10.1186/s12960-021-00694-w (PMC8627087; doi:10.1186/s12960-021-00694-w)
Supplement: Supplementary file 1 — Additional file 1: Table S1. Health occupations classed as part of the SRMNAH workforce. Table S2. List of WHO Member States by World Bank income group, 2020. Table S3. List of WHO Member States by WHO region, 2020. [file 12960_2021_694_MOESM1_ESM.docx]

**The State of the World’s Midwifery 2021 report: findings to drive global policy and practice**

**Supplementary tables**

**Table S1: Health occupations classed as part of the SRMNAH workforce**

| **Occupation** | **ISCO code** | **Examples** |
| --- | --- | --- |
| Midwifery professionals | 2222 | Professional midwife, technical midwife, midwife |
| Nursing professionals with midwifery training | 2221 | Nurse-midwife, perinatal nurse, maternity nurse |
| Nursing professionals | 2221 | Clinical nurse consultant, district nurse, nurse anaesthetist, nurse practitioner, operating theatre nurse, professional nurse, public health nurse, specialist nurse |
| Midwifery associate professionals | 3222 | Assistant midwife, auxiliary midwife |
| Nursing associate professionals with midwifery training | 3221 | Auxiliary nurse-midwife |
| Nursing associate professionals | 3221 | Assistant nurse, associate professional nurse, enrolled nurse, practical nurse, auxiliary nurse |
| Obstetricians and gynaecologists | 2212 | Obstetrician, gynaecologist |
| Paediatrician practitioners | 2212 | Paediatrician |
| General medical practitioners | 2211 | Family medical practitioner, general practitioner, medical doctor (general), medical officer (general), physician (general), primary care physician |
| Paramedical practitioners | 2240 | Advanced care paramedic, clinical officer (paramedical), feldscher, primary care paramedic, surgical technician, MEDEX |
| Medical assistants | 3256 | Clinical assistant, medical assistant |
| Community health workers | 3253 | Community health aide, community health promoter, community health worker, village health worker |

* This list is not comprehensive; other occupation groups include dieticians and nutritionists, anaesthetists, pharmacists and physiotherapists. However, these groups (a) are considered necessary for the delivery of the essential SRMNAH interventions listed in the *Global Strategy for Women’s, Children’s and Adolescents’ Health*, and (b) are identified in WHO’s National Health Workforce Accounts platform.
Source: adapted from the International Labour Organization’s International Standard Classification of Occupations ISCO-08.^[[1]](#footnote-1)^

*A note on professional and associate professional nurses and midwives.*

The key difference between a professional nurse or midwife and an associate professional relates to professional autonomy. A professional assumes responsibility for the planning and management of care either autonomously or as part of a team, whereas an associate professional provides basic care and generally works under the supervision or in support of professionals.

**Table S2: List of WHO Member States by World Bank income group, 2020**

| **Income group** | **Countries** |
| --- | --- |
| High (n=61) | Andorra, Antigua & Barbuda, Australia, Austria, Bahamas, Bahrain, Barbados, Belgium, Brunei Darussalam, Canada, Chile, Cook Islands*, Croatia, Cyprus, Czech Republic, Denmark, Estonia, Finland, France, Germany, Greece, Hungary, Iceland, Ireland, Israel, Italy, Japan, Kuwait, Latvia, Lithuania, Luxembourg, Malta, Mauritius, Monaco, Nauru, Netherlands, New Zealand, Norway, Oman, Palau, Panama, Poland, Portugal, Qatar, Republic of Korea, Romania, Saint Kitts & Nevis, San Marino, Saudi Arabia, Seychelles, Singapore, Slovakia, Slovenia, Spain, Sweden, Switzerland, Trinidad & Tobago, United Arab Emirates, United Kingdom, United States of America, Uruguay. |
| Upper middle (n=55) | Albania, Argentina, Armenia, Azerbaijan, Belarus, Belize, Bosnia & Herzegovina, Botswana, Brazil, Bulgaria, China, Colombia, Costa Rica, Cuba, Dominica, Dominican Republic, Ecuador, Equatorial Guinea, Fiji, Gabon, Georgia, Grenada, Guatemala, Guyana, Indonesia, Iran, Iraq, Jamaica, Jordan, Kazakhstan, Lebanon, Libya, Malaysia, Maldives, Marshall Islands, Mexico, Montenegro, Namibia, Niue*, North Macedonia, Paraguay, Peru, Russian Federation, Saint Lucia, Saint Vincent & the Grenadines, Samoa, Serbia, South Africa, Suriname, Thailand, Tonga, Turkey, Turkmenistan, Tuvalu, Venezuela. |
| Lower middle (n=49) | Algeria, Angola, Bangladesh, Benin, Bhutan, Bolivia, Cabo Verde, Cambodia, Cameroon, Comoros, Congo, Côte d’Ivoire, Djibouti, Egypt, El Salvador, Eswatini, Ghana, Honduras, India, Kenya, Kiribati, Kyrgyzstan, Lao People’s Democratic Republic, Lesotho, Mauritania, Micronesia (Federated States of), Mongolia, Morocco, Myanmar, Nepal, Nicaragua, Nigeria, Pakistan, Papua New Guinea, Philippines, Republic of Moldova, Sao Tome & Principe, Senegal, Solomon Islands, Sri Lanka, Tanzania, Timor-Leste, Tunisia, Ukraine, Uzbekistan, Vanuatu, Viet Nam, Zambia, Zimbabwe. |
| Low (n=29) | Afghanistan, Burkina Faso, Burundi, Central African Republic, Chad, Democratic People’s Republic of Korea, Democratic Republic of the Congo, Eritrea, Ethiopia, Gambia, Guinea, Guinea-Bissau, Haiti, Liberia, Madagascar, Malawi, Mali, Mozambique, Niger, Rwanda, Sierra Leone, Somalia, South Sudan, Sudan, Syrian Arab Republic, Tajikistan, Togo, Uganda, Yemen. |

* This country was not included in the World Bank income group classification, so an assumption was made based on gross national income.

**Table S3: List of WHO Member States by WHO region, 2020**

| **WHO region** | **Countries** |
| --- | --- |
| Africa (n=47) | Algeria, Angola, Benin, Botswana, Burkina Faso, Burundi, Cabo Verde, Cameroon, Central African Republic, Chad, Comoros, Congo, Côte d’Ivoire, Democratic Republic of the Congo, Equatorial Guinea, Eritrea, Eswatini, Ethiopia, Gabon, Gambia, Ghana, Guinea, Guinea-Bissau, Kenya, Lesotho, Liberia, Madagascar, Malawi, Mali, Mauritania, Mauritius, Mozambique, Namibia, Niger, Nigeria, Rwanda, Sao Tome & Principe, Senegal, Seychelles, Sierra Leone, South Africa, South Sudan, Tanzania, Togo, Uganda, Zambia, Zimbabwe. |
| Americas (n=35) | Antigua & Barbuda, Argentina, Bahamas, Barbados, Belize, Bolivia, Brazil, Canada, Chile, Colombia, Costa Rica, Cuba, Dominica, Dominican Republic, Ecuador, El Salvador, Grenada, Guatemala, Guyana, Haiti, Honduras, Jamaica, Mexico, Nicaragua, Panama, Paraguay, Peru, Saint Kitts & Nevis, Saint Lucia, Saint Vincent & the Grenadines, Suriname, Trinidad & Tobago, United States of America, Uruguay, Venezuela. |
| Eastern Mediterranean (n=21) | Afghanistan, Bahrain, Djibouti, Egypt, Iran, Iraq, Jordan, Kuwait, Lebanon, Libya, Morocco, Oman, Pakistan, Qatar, Saudi Arabia, Somalia, Sudan, Syrian Arab Republic, Tunisia, United Arab Emirates, Yemen. |
| Europe (n=53) | Albania, Andorra, Armenia, Austria, Azerbaijan, Belarus, Belgium, Bosnia & Herzegovina, Bulgaria, Croatia, Cyprus, Czech Republic, Denmark, Estonia, Finland, France, Georgia, Germany, Greece, Hungary, Iceland, Ireland, Israel, Italy, Kazakhstan, Kyrgyzstan, Latvia, Lithuania, Luxembourg, Malta, Monaco, Montenegro, Netherlands, North Macedonia, Norway, Poland, Portugal, Republic of Moldova, Romania, Russian Federation, San Marino, Serbia, Slovakia, Slovenia, Spain, Sweden, Switzerland, Tajikistan, Turkey, Turkmenistan, Ukraine, United Kingdom, Uzbekistan. |
| South-East Asia (n=11) | Bangladesh, Bhutan, Democratic People’s Republic of Korea, India, Indonesia, Maldives, Myanmar, Nepal, Sri Lanka, Thailand, Timor-Leste. |
| Western Pacific (n=27) | Australia, Brunei Darussalam, Cambodia, China, Cook Islands, Fiji, Japan, Kiribati, Lao People’s Democratic Republic, Malaysia, Marshall Islands, Micronesia (Federated States of), Mongolia, Nauru, New Zealand, Niue, Palau, Papua New Guinea, Philippines, Republic of Korea, Samoa, Singapore, Solomon Islands, Tonga, Tuvalu, Vanuatu, Viet Nam. |

1. International Labour Organization. International standard classification of occupations (ISCO). Geneva: International Labour Organization; 2008. [Available from: <https://www.ilo.org/public/english/bureau/stat/isco/isco08/index.htm>, accessed 6 February 2021]. [↑](#footnote-ref-1)
